# Extracellular vesicles derived from human umbilical cord mesenchymal stem cells alleviate osteoarthritis of the knee in mice model by interacting with METTL3 to reduce m6A of NLRP3 in macrophage

**DOI:** 10.1186/s13287-022-03005-9

**Published:** 2022-07-16

**Authors:** Hao Zhou, Xun Shen, Chen Yan, Wu Xiong, Zemeng Ma, Zhenggang Tan, Jinwen Wang, Yao Li, Jiuxiang Liu, Ao Duan, Feng Liu

**Affiliations:** 1 Department of Orthopedics, the First Affiliated Hospital of Nanjing Medical University, Nanjing, 210029 Jiangsu China; 2 Department of Orthopedics, Sir Run Run Hospital, Nanjing Medical University, Nanjing, 211100 Jiangsu China; 3 Department of Orthopedics, the First People’s Hospital of Lianyungang, Nanjing Medical University, Lianyungang,, 222002 Jiangsu China; 4 Key Laboratory of Immune Microenvironment and Disease, Department of Immunology, Nanjing Medical University, Nanjing, 211100 China

**Keywords:** Osteoarthritis, Human umbilical cord mesenchymal stem cells, Extracellular vesicles (EVs), miR-1208, METTL3, m6A, NLRP3

## Abstract

**Background:**

Osteoarthritis (OA) is a prevalent degenerative joint disease that not only significantly impairs the quality of life of middle-aged and elderly individuals but also imposes a significant financial burden on patients and society. Due to their significant biological properties, extracellular vesicles (EVs) have steadily received great attention in OA treatment. This study aimed to investigate the influence of EVs on chondrocyte proliferation, migration, and apoptosis and their protective efficacy against OA in mice.

**Methods:**

The protective impact of EVs derived from human umbilical cord mesenchymal stem cells (hucMSCs-EVs) on OA in mice was investigated by establishing a mouse OA model by surgically destabilizing the medial meniscus (DMM). Human chondrocytes were isolated from the cartilage of patients undergoing total knee arthroplasty (TKA) and cultured with THP-1 cells to mimic the in vivo inflammatory environment. Levels of inflammatory factors were then determined in different groups, and the impacts of EVs on chondrocyte proliferation, migration, apoptosis, and cartilage extracellular matrix (ECM) metabolism were explored. N6-methyladenosine (m6A) level of mRNA and methyltransferase-like 3 (METTL3) protein expression in the cells was also measured in addition to microRNA analysis to elucidate the molecular mechanism of exosomal therapy.

**Results:**

The results indicated that hucMSCs-EVs slowed OA progression, decreased osteophyte production, increased COL2A1 and Aggrecan expression, and inhibited ADAMTS5 and MMP13 overexpression in the knee joint of mice via decreasing pro-inflammatory factor secretion. The in vitro cell line analysis revealed that EVs enhanced chondrocyte proliferation and migration while inhibiting apoptosis. METTL3 is responsible for these protective effects. Further investigations revealed that EVs decreased the m6A level of NLRP3 mRNA following miR-1208 targeted binding to METTL3, resulting in decreased inflammatory factor release and preventing OA progression.

**Conclusion:**

This study concluded that hucMSCs-EVs inhibited the secretion of pro-inflammatory factors and the degradation of cartilage ECM after lowering the m6A level of NLRP3 mRNA with miR-1208 targeting combined with METTL3, thereby alleviating OA progression in mice and providing a novel therapy for clinical OA treatment.

## Introduction

Osteoarthritis (OA) is a prevalent joint degenerative disease primarily affecting middle-aged and elderly individuals, causing joint enlargement and deformity, joint pain, limited functional activities, seriously affecting patients' quality of life, and imposing a significant economic burden on families and society [1–6]. The most prevalent joint affected by OA is the knee, and the population affected by knee OA is growing as the Chinese population ages [4]. Gender, age, degeneration, inflammation, extracellular matrix (ECM) degradation, obesity, injury, improper knee alignment, genetic susceptibility, and other factors have all been implicated in the knee OA etiology [2, 6–9]. The involvement of low-grade inflammation in OA incidence and progression is largely recognized among them [6, 9]. The inflammation causes abnormal cartilage ECM metabolism, characterized by a decrease in type II collagen alpha 1 (COL2A1) and the structural proteoglycan Aggrecan and an increase in cartilage matrix-degrading enzymes such as a disintegrin-like and metalloproteinase domain with thrombospondin-1 motifs 5 (ADAMTS5) and matrix metalloproteinase 13 (MMP13), resulting in cartilage ECM degradation and further aggravating OA progression [10, 11]. Although drug therapy, physical therapy, and surgical treatment are currently available for knee OA [2, 12], few treatments can reduce or delay OA development.

Inflammation is a key factor in OA onset and progression [6, 13]. Pro-inflammatory factors such as IL-1β, IL-18, and TNF-α may accelerate the course of OA by increasing cartilage ECM degradation [14, 15]. The primary cells regulating inflammation in OA are macrophages, which release various pro-inflammatory factors [16, 17] and cytokines such as IL-1β and IL-18 by activating the nod-like receptor pyrin domain 3 inflammasomes (NLRP3) [18, 19]. In recent years, inflammation in OA relating to NLRP3 activation has also been intensively researched [20]. Inactivated caspase-1 is cleaved into activated caspase-1 after the combination of NLRP3 inflammasome, and activated caspase-1 cleaves pro-inflammatory factors IL-1β and IL-18 into activated IL-1β and IL-18, which play roles in inflammation, causing ECM degradation, and promoting OA occurrence and development [21–23]. Inhibiting NLRP3 activation in macrophages is thus envisaged to prevent OA onset and progression.

N6-methyladenosine (m6A), the most common post-transcriptional modification of mRNA within eukaryotic cells [24], determines the stability, cleavage, transfer, and translation efficiency of mRNA [25]. Several studies have found that m6A is linked to inflammation [26–28]. M6A is a dynamic, reversible modification process, which can be increased by methyltransferases or decreased by demethyltransferases [24, 25, 29]. Methyltransferase-like 3 (METTL3), the most common mRNA methyltransferase in eukaryotic cells, has been linked to OA development [30–33]. By knocking down METTL3, OA progression and subsequent degradation of ECM can be slowed, and the apoptosis rate of chondrocytes is reduced [31, 32]. However, the specific mechanism by which METTL3 alleviates OA has not been clearly elucidated.

Stem cell therapy has recently emerged as an innovative and effective treatment strategy, and mesenchymal stem cells (MSCs) have attracted significant interest in degenerative diseases due to their self-renewal ability and multi-differentiation potential [34–37]. It has been reported that MSCs derived from the inferior fat pad and MSCs derived from the synovium can alleviate knee OA progression [38, 39]. Human umbilical cord mesenchymal stem cells (hucMSCs) offer the advantages of facile extraction and expansion, cost-effectiveness, atraumatic collection process, high cell content, and minimal risk of infection, making them a suitable therapeutic approach [36, 37]. Its therapeutic applicability is limited due to the low number of donor umbilical cord stem cells, possible chromatin variation, and immunological exclusion [36]. Extracellular vesicles (EVs), as one of the major extracellular signaling pathways, have been linked to various disorders and are engaged in various physiological processes [40, 41]. EVs can be secreted by almost any cell. EVs generated from stem cells have no other adverse effects, such as immunological exclusion, tumorigenicity, etc., compared to stem cells [41, 42]. Wu et al. reported that EVs derived from inferior fat pad MSCs could alleviate OA through miR-100-5p, and Xu et al. reported that synovial MSCs-EVs could promote cartilage regeneration [38, 39]. Previous research has revealed that human umbilical cord stem cells have therapeutic potential for OA [43]. However, there is no literature on the efficacy of hucMSCs-EVs for OA. Therefore, in this study, we intensively investigated the efficacy of hucMSCs-EVs on OA.

This study investigated the interplay effects of hucMSCs-EVs to inhibit the release of pro-inflammatory factors in macrophages, degradation of cartilage ECM, promotion of chondrocyte proliferation and migration, and inhibition of chondrocyte apoptosis via NLRP3 after interacting with METTL3 mRNA via miR-1208. The approach is envisaged to slow down OA progression and provide a potential foundation for the clinical treatment of knee OA.

## Methods and materials

### Cell culture

Human articular chondrocytes were acquired from patients undergoing total knee arthroplasty (TKA) surgery, and all patients were briefly informed about the objective of the investigation, and signed written consent was obtained. Only specimens from patients who had TKA for knee OA were collected; patients who had surgery for traumatic knee OA, rheumatoid arthritis, or infectious arthritis were excluded from the study. The isolation protocol has already been detailed previously [21]. After obtaining the cartilage tissue, it was washed twice with phosphate-buffered saline (PBS), adding 1% penicillin/streptomycin (P/S, Gibco). The cartilage tissue was then sliced into fragments around 3 × 3 mm in size with a sterile scalpel before being digested for 30 min in 2% trypsin (Gibco). The trypsin was then discarded, and the digestion was stopped by adding Dulbecco's Modified Eagle Medium/Nutrient Mixture F-12 (DMEM/F12, Gibco) containing 20% fetal bovine serum (FBS) and 1% P/S. The digested material was then incubated for 16 h with collagenase type II (Gibco) at a 2 mg/mL ratio for further digestion. Following digestion, the chondrocytes were filtered through a cell sieve and digested again for 15 min with DNA enzyme (Gibco). After centrifuging the cells three times to acquire primary chondrocytes, they were resuspended in DMEM/F12 with 20% FBS and 1% P/S, evenly seeded in a culture dish, and incubated at 37 °C under a 5% CO_2_ atmosphere for cultivation for the next experiment.

Cyagen Biotechnology Inc. provided the passage of two generations of hucMSCs (130606L01, Cyagen Biotechnology Inc., Guangzhou, China). hucMSCs were cultured in a low-glucose DMEM (Gibco) containing 10% FBS and 1% P/S, with the culture medium changed every 3–4 days. When the cells were 80–90% confluent, performing cell passage and the 3rd and 4th passages of hucMSCs were employed for subsequent investigations.

The American Type Culture Collection (Nr. TIB-202) provided THP-1, a monocytic cell line derived from human peripheral blood. THP-1 cells were cultured at 37 °C under 5% CO_2_ atmosphere in an incubator using RPMI 1640 medium (Gibco) containing 10% FBS and 1% P/S.

### Cell transfection

Cell transfection was conducted using adherent THP-1 cells, where the phorbol myristate acetate (PMA, P1585, Sigma-Aldrich) induced THP-1 cell adhesion. The siMETTL3-RNA (RiboBio), siAgo2-RNA (Ruibo Biotech, China), and Lipofectamine 2000 (Invitrogen) were transfected into adherent THP-1 cells. The siMETTL3-RNA sequence is 5ʹ-GCACTTGGATCTACGGAAT-3ʹ.

### Isolation of EVs and endocytosis of EVs into macrophages

When hucMSCs reached 90% confluence, EVs were extracted by washing the cells twice with PBS, followed by re-culturing and incubation for 48 h in the presence of EV-free serum medium. Following supernatant extraction, EVs were isolated using differential centrifugation, viz. centrifugation at 2000 × g for 10 min to remove cell debris; 10,000 × g for 10 min to remove apoptotic bodies, and 100,000 × g for 90 min to remove the supernatant and collect impurities-containing EVs. The collected EVs were then washed twice by suspending them in 20 mL PBS and centrifuging them again at 100,000 × g for 90 min. EVs were then suspended in 50 mL PBS and stored at − 80 °C until further use.

The size distribution of EVs was determined using a NanoSight NS300 (Malvern Panalytical Ltd.), and EVs morphology was examined by employing transmission electron microscopy (Tecnai 12 transmission electron microscope, Philips, Netherland).

To determine whether THP-1 cells may endocytose EVs, EVs were labeled with Dil solution (4 mg/mL, Sigma) and then co-cultured with THP-1 cells for 24 h. After washing with PBS, THP-1 cells were stained for 15 min with 4′,6-diamidino-2-phenylindole (DAPI, Thermo). A fluorescence microscope was used to view and photograph the result (Zeiss, Germany).

### Knee osteoarthritis model

Healthy male C57BL/6 mice (eight weeks) were purchased from the Animal Resource Center of the Faculty of Medicine, Nanjing Medical University, while Yunzi Chen Laboratory provided NLRP3^−/−^ mice. All mice were housed and handled as per defined procedures of the Animal Resource Center at Nanjing Medical University's Faculty of Medicine. OA knee mice model was created via surgically destabilizing the medial meniscus (DMM). In brief, mice were randomly divided into two groups labeled sham and OA groups. The sham group mice were anesthetized intraperitoneally with 35 mg/kg pentobarbital before sham surgery. The skin and joint capsule were incised, and then, the joint capsule and skin were sutured sequentially. The medial meniscus and anterior cruciate ligament (ACL) were removed in OA group, and the joint capsule and skin were sutured. After surgery, ibuprofen was given to each mouse to relieve pain, and penicillin was provided to prevent infection. Three days after surgery, the mice were forced to exercise for one hour per day for six weeks on a customized treadmill.

### Intra-articular injection of EVs and antagomiR

The mice with DMM surgery were randomly separated into two groups, while those with the sham procedure were assigned to the sham group. Mice were then grouped into sham group, PBS group, and hucMSCs-EVs group. A total of 10 μL PBS or hucMSCs-EVs (10^11^ particles /mL) were injected intra-articularly twice a week to the mice in each group. Each mouse was made to run for 1 h per day for six weeks on a special treadmill.

In terms of antagomiR intra-articular delivery, mice were randomly divided into EVs + antagomiR-NC and EVs + antagomiR-1208. For three weeks before surgery, mice received twice-weekly intra-articular injections of either antagomiR-NC (10 μL, 200 nmol/mL) or antagomiR-1208 (10 μL, 200 nmol/mL). Following that, DMM surgery was performed on all mice. Following the surgery, mice received intra-articular injections of EVs + antagomiR-NC (10 μL, 200 nmol/mL) or EVs + antagomiR-1208 (10 μL, 200 nmol/mL) twice a week for six weeks. For six weeks, all mice were made to run for 1 h per day.

### Safranin O and Fast Green staining

Decalcified, dehydrated, paraffin-embedded mice knee samples were sliced into 5-μm-thick sections for Safranin O and Fast Green staining. After deparaffinization with xylene and 100% ethanol, the sections were stained with Safranin O and Fast Green FCF Stain Kit (Solarbio) according to manufacturer's instructions. Sections were stained for 2 min with Fast Green dye solution and then placed in water for differentiation for 5 min before being counterstained with Safranin O dye solution for 10 min, followed by cleaning with xylene for 5 min. The stained sections were examined and photographed using a microscope (Olympus, Japan). Stained sections were scored for the Osteoarthritis Research Society International (OARSI) score [44].

### Micro-computed tomography (micro-CT) joint imaging

The structural changes in the knee joint of mice were observed using SkyScan 1176 Micro-CT equipment (Bruker, Billerica, MA, USA) with an 8 µm scanning resolution. SkyScan volumetric NRecon reconstruction software version 1.6 was used to reconstruct 3D images (Bruker, Billerica, MA, USA).

The knees of mice in different groups were fixed on a Micro-CT platform for CT scanning after anesthesia by pentobarbital peritoneal injection. Osteophytes were then scored based on 3D reconstructed CT scan images [21].

### Immunofluorescence staining

Tissue slices were prepared for immunofluorescence staining as described in the previous section. After the sections were deparaffinized with xylene and ethanol, antigen retrieval was performed in an oven at 37 °C for 1 h in 2 mg/mL of trypsin liquid. The slices were then immersed in PBS for 5 min three times before being blocked with 10% goat serum at room temperature for 2 h and treated with various primary antibodies at 4 °C for 8 h. The sections were then washed three times with PBST solution before being incubated with homologous Alexa Fluor 488 or 555 secondary antibodies for 1 h at room temperature in the dark. The sections were viewed and photographed under a fluorescence microscope (Olympus, Japan) after the nucleus was stained with DAPI. Primary antibodies used in the experiments included antibodies against COL2A1 (1: 200, Abcam, ab34712), antibodies against Aggrecan (1:200, Proteintech, 13,880–1-AP), antibodies against ADAMTS5 (1:200; Abcam, ab182795), and antibodies against MMP13 (1:200, Proteintech, 18,165–1-AP).

### Enzyme-linked immunosorbent assay (ELISA)

ELISA was used to quantify the amounts of inflammatory factors such as IL-1β, IL-18, and TNF-α in mice knee and cell supernatants. After homogenizing mice knee synovial tissue and synovial fluid with radioimmunoprecipitation assay (RIPA) lysate for 30 min, the supernatants were collected, and the levels of inflammatory factors in the supernatants were evaluated using ELISA kit (Cloud-Clone Corp, SEA563Hu, SEA064Hu, and SEA133Hu) according to instructions. ELISA kits were also used to detect inflammatory components in the cell supernatant.

### Proliferation assessment of chondrocytes

EdU kit was used to test chondrocyte proliferative capability. A cell co-culture system was created by employing a 24-well transwell chamber (pore size: 0.4 μm, Corning) to evaluate chondrocyte proliferative potential. First, the chondrocytes were seeded into 24-well plates and cultured to around 90% cell density for future use. THP-1 cells were seeded in the transwell upper chamber followed by using lipopolysaccharide (LPS) and Nigericin (Nig) treatment after cell attachment with phorbol myristate acetate (PMA, P1585, Sigma-Aldrich) to stimulate THP-1 cells to differentiate into macrophages. The co-culture system was then established by inserting transwell chambers into chondrocyte-adherent 24-well plates and incubating for 24 h in a medium containing EdU (10 μM). The proliferation rate was evaluated using BeyoClick™EdU-594 Cell Proliferation Detection Kit (Beyotime) according to manufacturer's instructions. Fluorescence microscopy and flow cytometry were used to examine cell proliferation in each group.

### Apoptosis assessment of chondrocytes

The effect of hucMSCs-EVs on chondrocyte apoptosis was investigated using a TUNEL kit and flow cytometry. Chondrocytes were uniformly seeded in the lower chamber in the co-culture system, while macrophages were cultivated in the upper chamber and either treated with hucMSCs-EVs or not. Following 24 h of incubation, cells in each group were stained with the Annexin V-FITC/PI Apoptosis Detection Kit (Vazyme) according to manufacturer's instructions. The difference in apoptosis rate between groups was determined by flow cytometry. When using TUNEL kit, each group's chondrocytes were stained using TUNEL Bright Green Apoptosis Detection Kit (Vazyme) according to manufacturer's instructions, and apoptosis was detected and photographed using a fluorescence microscope.

### Migration assessment of chondrocytes

The transwell test and cell scratch area healing assay were performed to determine chondrocyte migration. In the cell scratch area healing assay, chondrocytes were seeded at 100% density in 6-well plates. A sterile pipette tip (200 μL) was utilized to scratch the cell layer. After scratching, the cells were gently washed three times with PBS solution and replenished with EV-containing media or without EVs. To assess the influence of EVs on chondrocyte migration, the cells were observed and photographed at intervals of 0, 24, and 48 h. Before scratch studies, the cells were treated with mitomycin (1 μg/mL) for 1 h to eliminate cell proliferation effects [45]. For the transwell assay, chondrocytes were seeded in the upper chamber and macrophages in the lower chamber for 24 h with or without EVs. After incubation, the transwell upper chamber was fixed for 20 min with 4% paraformaldehyde (PFA), and the upper chamber's upper surface was wiped to eliminate non-migrated cells. After staining with 0.5% crystal violet for 30 min, the chamber was washed three times with PBS solution. Then, a microscope was used to observe the migration of chondrocytes in each group.

### Measurement of m6A modification

The m6A levels in RNA were determined using a reported method [46]. Briefly, total RNA was isolated from cells and treated with DNase. The total m6A RNA was detected using an m6A RNA methylation assay kit (Abcam, ab185912) according to manufacturer's protocol. The assay wells were coated with 200 ng RNA. The appropriate dilutions of capture and detection antibody solutions were then applied to the test wells. m6A levels were then quantified calorimetrically by reading the absorbance at 450 nm and then calculating the m6A level in RNA using the standard curve.

### Western blot analysis

The protein expression of cells in different treatment groups was determined by Western blotting technique. Protein lysate samples were obtained in each group following treatment with RIPA lysate. BCA technique was used to determine the protein concentration in the samples. Protein samples were separated by electrophoresis on an SDS–polyacrylamide gel and then electro-transferred to a polyvinylidene fluoride (PVDF) membrane. PVDF membranes were blocked in 5% BSA liquid for 2 h at room temperature before incubating with the primary antibodies overnight at 4 °C. After washing with TBST solution, the secondary antibody was incubated at room temperature for 1 h. Again, washing was conducted three times with TBST solution, and then the protein bands were recorded using an ECL reagent and a Tanon 5200 chemiluminescence image analysis system. The Image J software was used to assess gray values. Primary antibodies used in this experiment included antibodies against COL2A1 (1:2000, Abcam, ab188570), antibodies against Aggrecan (1:2000, Proteintech, 13,880–1-AP), antibodies against ADAMTS5 (1:2000, Abcam, ab182795), antibodies against MMP13 (1:2000, Proteintech, 18,165–1-AP), antibodies against CD9 (1:2000; Abcam; ab223052), antibodies against CD63 (1:2000; Abcam; ab134045), antibodies against Alix (1:1500; Proteintech; 12,422–1-AP), antibodies against TSG101 (1:2000; Proteintech; 14,497–1-AP), antibodies against Calnexin (1:2000; Proteintech; 10,427–2-AP), antibodies against Tubulin (1:2000; Proteintech; 10,094–1-AP), antibodies against Ago2 (1:2000; Abcam; ab186733), antibodies against NLRP3 (1:1000, Adipogen, AG-20B-0014), antibodies against METTL3 (1:2000, Proteintech; 15,073–1-AP), antibodies against IL-1β (1:1000, R&D Systems, AF-401-NA), antibodies against Ubiquitin (1:1000, Santa Cruz, sc-8017), and antibodies against caspase-1 (1:1000, Abcam, ab108362).

### miRNA target prediction analysis

TargetScan (http://www.targetscan.org/vert 80/), GeneCards (https://www.genecards.org/), and miRWalk (http://mirwalk.umm.uni-heidelberg.de/) were used to predict the miRNA of target genes of interest, and the target miRNA was determined by the intersection of prediction results.

### miRNA microarray assay

Suri Medicine Company conducted miRNA arrays on hucMSCs-EVs (Nanjing, China). Fragmentation mixtures were crossed using Agilent-Human microRNA array 21.0. Additionally, microarray analysis was performed utilizing the Affymetrix (Santa Clara, CA, USA) miRNA 4.0 platform. Following the instructions, samples were labeled, and microarrays were hybridized and washed (Agilent Technologies Inc., Santa Clara, CA, USA).

### Luciferase reporter assay

GenScript (Nanjing, China) was used to create sequences corresponding to the 3′-UTR of METTL3 mRNA and containing the wild-type (WT) or mutant (MUT) miR-1208 binding sequences. The miR-1208 sequences were subsequently cloned at XbaI and FseI restriction sites in pGL3 luciferase reporter vector (Promega, Madison, WI, USA). The constructed pGL3 luciferase reporter vector was transfected into 293 T tool cells. A fluorescence microscope was used to observe the expression of fluorescently tagged genes in 293 T cells after 24 h of transfection. Dual-Luciferase® Reporter Assay System kit (E1910, Promega) was used to evaluate luciferase expression (E1910, Promega).

### Statistical analysis

All data are presented as mean ± standard deviation. GraphPad Prism 8.0 (GraphPad Software, Inc., San Diego, CA) and SPSS 26.0 were used to analyze data (IBM, Armonk, NY, USA). Before beginning data analysis, data distribution was checked for positivity and homogeneity of variance. Two different groups were compared by independent-sample t test, and multiple group comparisons were performed by ANOVA. Statistical significance was defined as a *p* < 0.05.

## Results

### Identification of hucMSCs-EVs and macrophages endocytose EVs

**Fig. 1:**
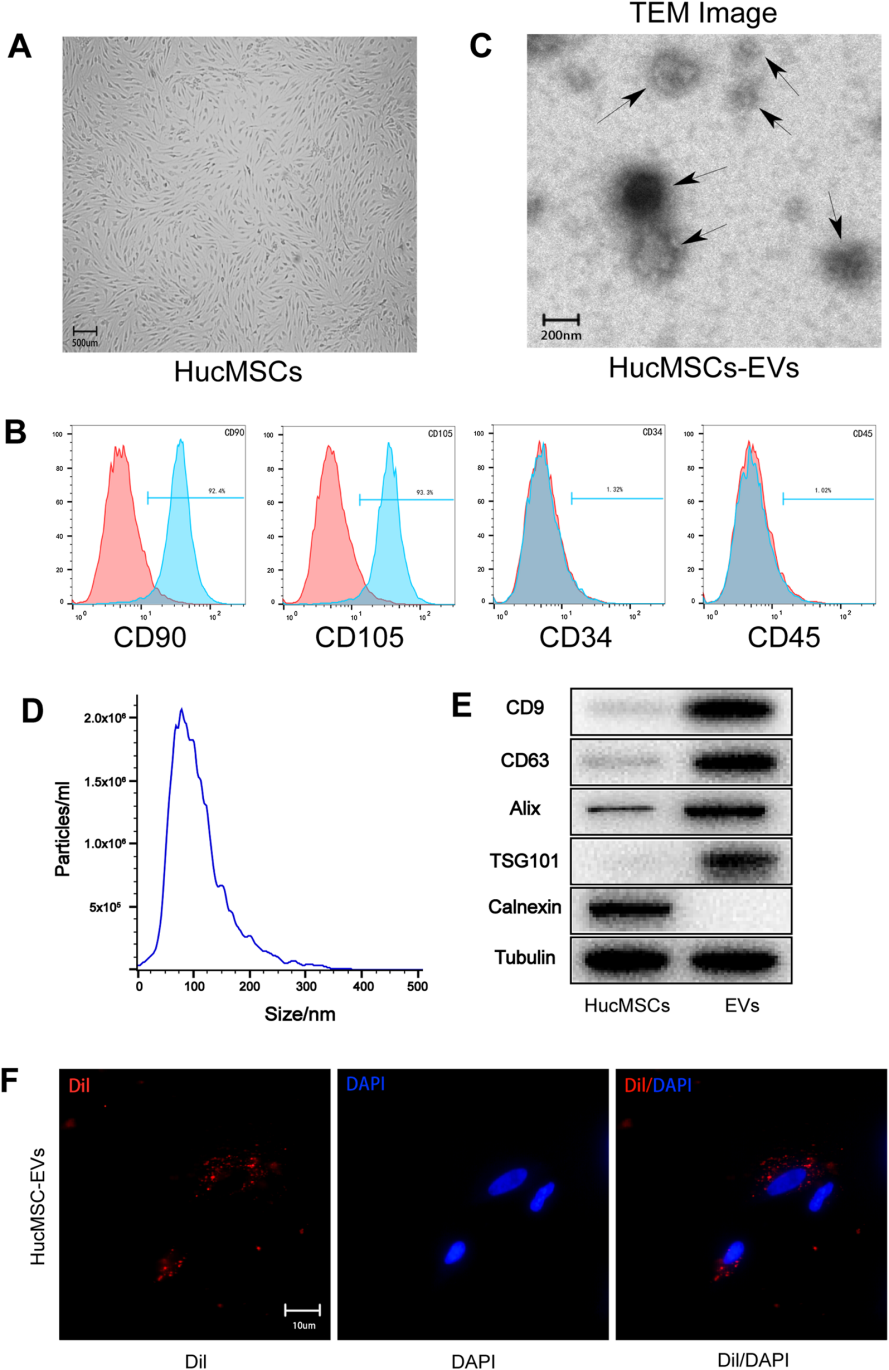
Identification of human umbilical cord MSCs and EVs. **A.** A typical spindle-like morphology of hucMSCs. **B.** Flow cytometry analysis showed that hucMSCs expressed specific markers on their surface. **C.** Transmission electron microscope image of hucMSCs-EVs. **D.** Particle size distribution of hucMSCs-EVs analyzed by NanoSight. **E.** EVs special markers (CD9, CD63, Alix, TSG101) analyzed by western blot. **F.** Macrophages endocytose EVs observed by fluorescence microscope

The hucMSCs 3^rd^ and 4^th^ passages were employed in this study. Following the cells reaching 80–90% confluence, they revealed a typical spindle-like MSC morphology (Fig. 1A). Flow cytometry was used to identify the surface antigens of hucMSCs, and they were shown to positively express CD90 and CD105 while negatively expressing CD34 and CD45 (Fig. 1B).

Ultracentrifugation was used to isolate EVs from the supernatant of hucMSCs. EVs revealed a cup-shaped or spherical morphology, with an average diameter of about 100 nm, according to transmission electron microscopy (TEM) (Fig. 1C). Like TEM, nanoparticle tracking analysis (NTA) examined the size distribution curve of EVs, which ranged in size from 50 to 200 nm (Fig. 1D). Western blot analysis revealed that particular EV markers such as CD9, CD63, Alix, and TSG101 were expressed in EVs, and the negative extracellular vesicle marker calnexin was absent (Fig. 1E).

Dil dye-labeled EVs were co-cultured with macrophages for 24 h to confirm that they could endocytose EVs. After labeling the nucleus with DAPI, macrophages were observed endocytosing EVs under a fluorescent microscope. The findings demonstrated that macrophages might endocytose hucMSCs-EVs (Fig. 1F).

### Effect of HucMSCs-EVs on knee OA progression and cartilage ECM degradation in mice model of DMM-induced OA

**Fig. 2:**
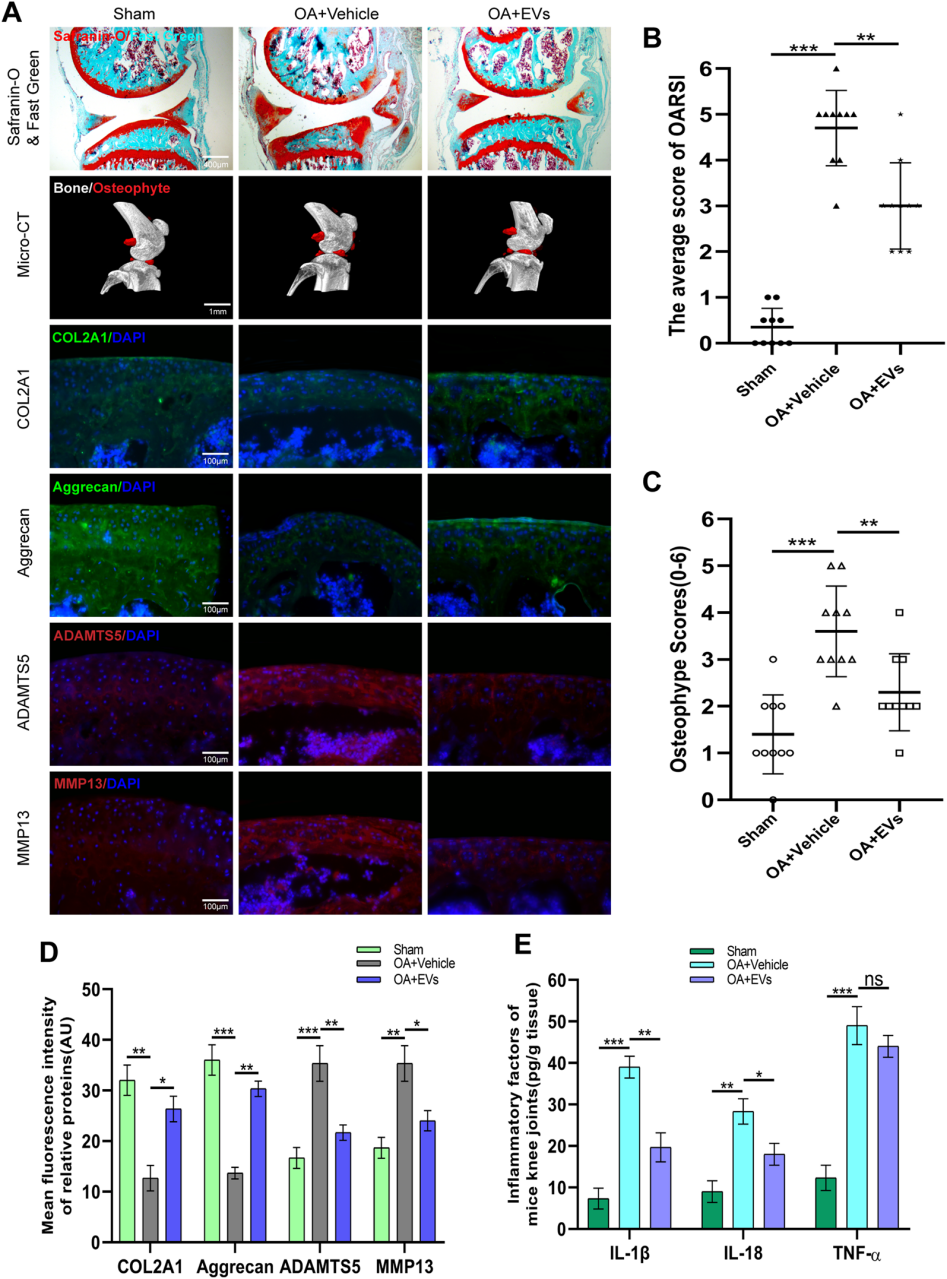
HucMSCs-EVs alleviate knee osteoarthritis in DMM-induced OA mice model. **A.** Safranin O and Fast Green staining of knee sections and micro-CT analysis of knee joint in mice with DMM-induced OA. Expression levels of COL2A1, Aggrecan, ADAMTS5, and MMP13 were determined by immunofluorescence staining. Scale bar = 400 μm for Safranin O and Fast Green staining; Scale bar = 1 mm for Micro-CT; Scale bar = 100 μm for immunofluorescence staining. **B.** OARIS scores of knee sections in each group (*n* = 10). **C.** Osteophyte score of each group (n = 10). **D.** Quantification of mean fluorescence intensity of COL2A1, Aggrecan, ADAMTS5, and MMP13 in each group (n = 10). **E.** Levels of IL-1β, IL-18, and TNF-α in mice knee tissue as determined by ELISA (n = 3). Data are presented as mean ± SD. ns, no significance; *p < 0.05; **p < 0.01; ***p < 0.001

After establishing DMM-induced knee OA mice model, different treatments were administered to separate groups, as detailed in the methods section. To evaluate knee cartilage, subchondral bone, and bone tissue structural alterations, sections of knee joint specimens from mice in different groups were stained with Safranin O and Fast Green. The results indicated that cartilage wear and subchondral bone exposure in the knee joint of mice treated with hucMSCs-EVs were considerably reduced compared to the vehicle group (Fig. 2A), indicating that hucMSCs-EVs have a protective effect against OA. Additionally, the Osteoarthritis Research Society International (OARSI) score in mice corroborated the protective impact of EVs against OA (Fig. 2B). Additionally, 3D reconstruction of mice's knees using Micro-CT revealed that osteophyte formation was significantly increased in the knees of vehicle-treated mice compared to the sham group, whereas osteophyte formation was decreased following EVs treatment (Fig. 2A), elucidating the protective effect of EVs against OA. Consistent with Micro-CT findings, mice treated with EVs had considerably lower osteophyte scores than mice treated with vehicles (Fig. 2C). These findings suggest that hucMSCs-EVs can partially reverse articular cartilage degradation and osteophyte formation in mice knees.

To examine ECM of knee cartilage in each group of mice, immunofluorescence staining was used to evaluate ECM proteins and matrix degradation-related proteases in each group. Immunofluorescence staining results indicated that COL2A1 and Aggrecan expression levels in the vehicle group were lower, whereas ADAMTS5 and MMP13 expression levels were higher than in the sham group. Notably, ECM proteins were not significantly reduced in EVs group, and the increase in matrix degradation-associated proteases was counteracted (Fig. 2A and D).

To determine the level of inflammatory factor expression in the knee joints of mice in each group, the collected knee tissues were subjected to ELISA analysis. ELISA results indicated that the vehicle group had significantly greater levels of IL-1β, IL-18, and TNF-α than the sham group, while EVs group had significantly lower levels of IL-1β and IL-18. Notably, there was no significant reduction in TNF-α expression in EVs group compared to the vehicle group (Fig. 2E). The above findings imply that hucMSCs-EVs can slow the course of knee OA in mice and limit cartilage ECM breakdown.

### Effect of HucMSCs-EVs knee OA in mice through NLRP3

**Fig. 3:**
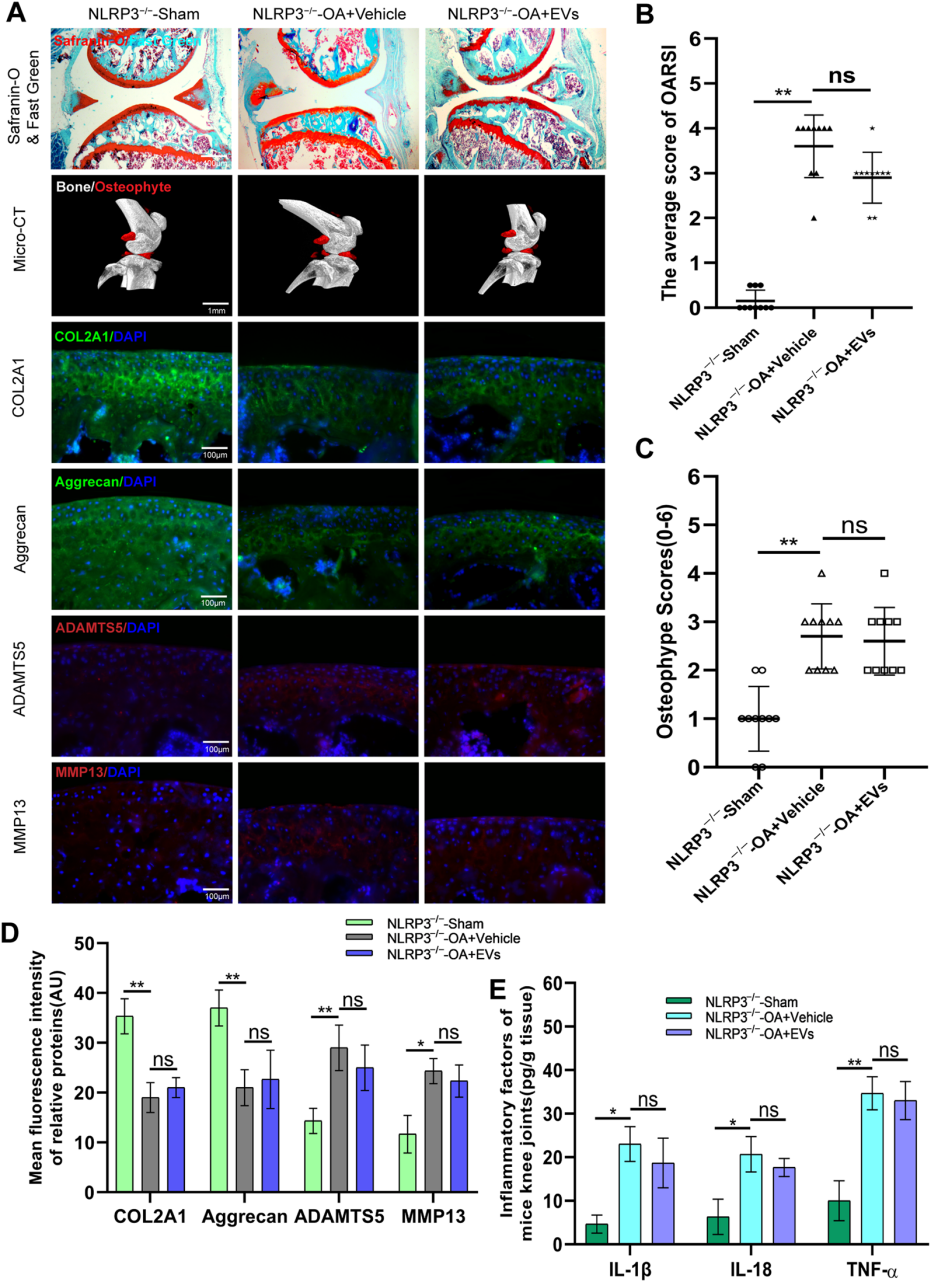
The protection effect of knee OA by hucMSCs-EVs was abolished in NLRP3^−/−^ mice. **A.** Safranin O and Fast Green staining of knee sections and micro-CT analysis of knee joint in NLRP3^−/−^ mice with DMM-induced OA. Expression levels of COL2A1, Aggrecan, ADAMTS5, and MMP13 were determined by immunofluorescence staining. Scale bar = 400 μm for Safranin O and Fast Green staining; Scale bar = 1 mm for Micro-CT; Scale bar = 100 μm for immunofluorescence staining. **B.** OARIS scores of knee sections in each group (*n* = 10). **C.** Osteophyte score of each group (n = 10). **D.** Quantification of mean fluorescence intensity of COL2A1, Aggrecan, ADAMTS5, and MMP13 in each group (n = 10). **E.** Levels of IL-1β, IL-18, and TNF-α in mice knee tissue as determined by ELISA (n = 3). Data are presented as mean ± SD. ns, no significance; *p < 0.05; **p < 0.01; ***p < 0.001

As an initiator of inflammation, NLRP3 inflammasome was the first to be investigated for its involvement in EV-mediated alleviation of OA. For validation, NLRP3^−/−^ mice were employed in this study. Modeling and group treatment were carried out as usual. Safranin O and Fast Green staining of specimen sections revealed that EVs had lost their protective function on the cartilage in the knee joints of NLRP3^−/−^ mice (Fig. 3A). OARSI score also confirmed the disappearance of cartilage protection function of EVs (Fig. 3B). Micro-CT images of mice knees displayed no significant difference in osteophyte formation between vehicle and EVs groups (Fig. 3A). The osteophyte score was consistent with the results of Micro-CT images (Fig. 3C).

Furthermore, immunofluorescence labeling of specimen sections revealed that vehicle and EVs groups had slightly lower levels of COL2A1 and Aggrecan than the sham group. Still, there was no significant difference between the two treatment groups (Fig. 3A and D). ADAMTS5 and MMP13 behaved similarly to COL2A1 and Aggrecan in that the results of vehicle and EVs groups were higher than the sham group, without significant difference between the two groups (Fig. 3A and D).

ELISA kits were used to detect the expression of inflammatory factors in the knee joints of mice in each group. ELISA results were comparable to the above. The levels of inflammatory factors IL-1β, IL-18, and TNF-α were statistically elevated in vehicle and EVs groups compared to the sham group, although IL-1β and IL-18 were not significantly decreased after EVs therapy (Fig. 3E). The preceding findings contradict those of a normal knee OA mouse model, in which the inflammatory factors decreased after treatment with EVs, implying that EVs exhibit anti-inflammatory effects via NLRP3 in OA mice model.

### Downregulation of METTL3 expression in macrophages by HucMSCs-EVs

**Fig. 4:**
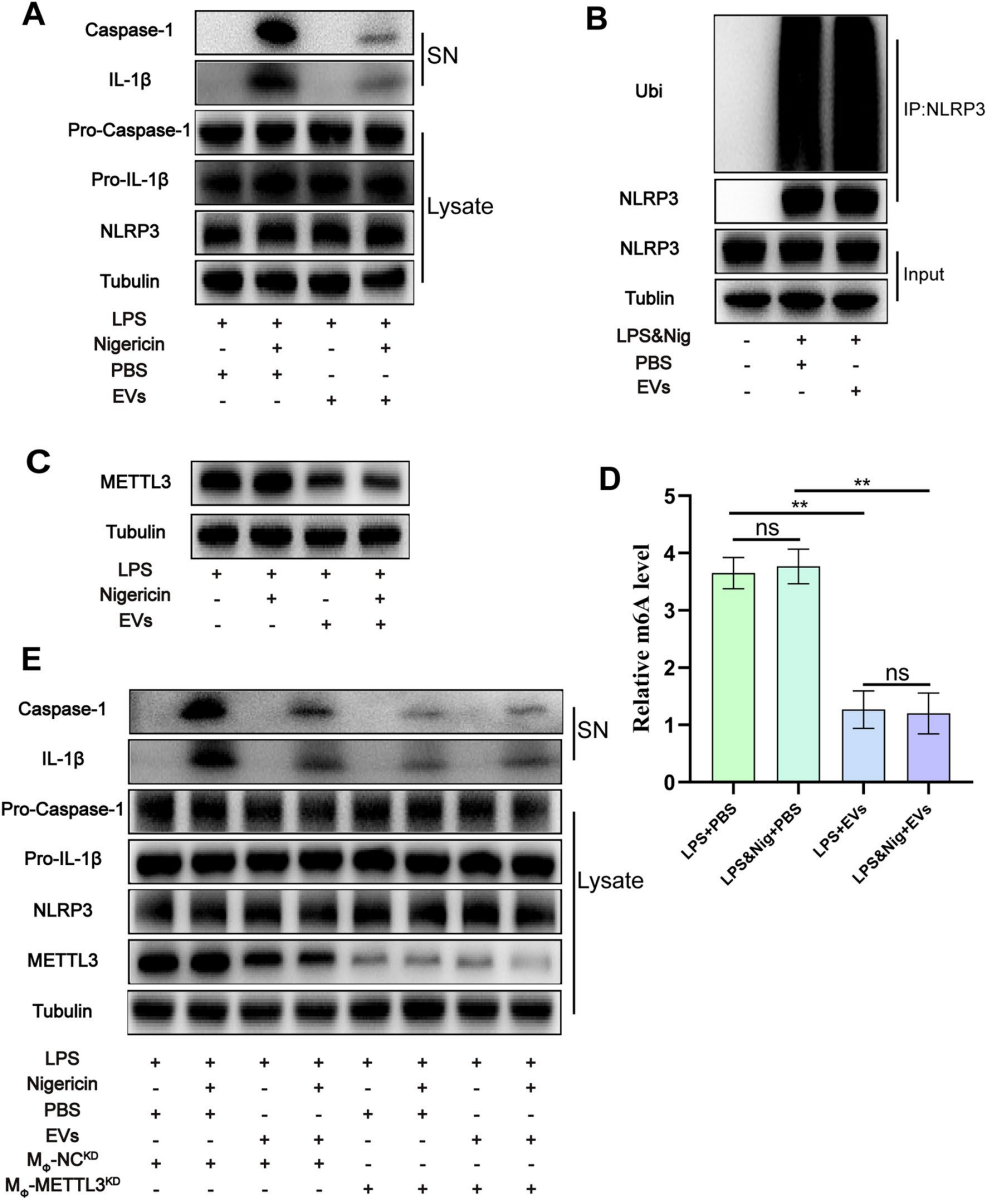
HucMSCs-EVs exert anti-inflammatory effect by downregulating METTL3 expression in macrophages. **A.** Western blot analysis of IL-1β and cleaved caspase-1 levels in culture SN and pro-IL-1β, pro-caspase-1, and NLRP3 levels in cell lysates in each group. **B.** NLRP3 ubiquitination was analyzed in THP-1 cells with different treatments. **C.** Western blot analysis of METTL3 levels in cell lysates in each group. **D.** The m6A level of RNA in each group (n = 3). **E.** Western blot analysis of IL-1β and cleaved caspase-1 levels in culture SN and pro-IL-1β, pro-caspase-1, NLRP3, and METTL3 levels in cell lysates in each group. Data are presented as mean ± SD. ns, no significance; *p < 0.05; **p < 0.01; ***p < 0.001

Inflammatory factors such as IL-1β and IL-18 were enhanced in the mice model of knee OA, and the anti-inflammatory impact of hucMSCs-EVs was reduced in NLRP3 − / − mice, demonstrating that EVs exert anti-inflammatory effects via NLRP3. After inflammasome creation, NLRP3 activates IL-1 via caspase-1 activation. To further explore the mechanism, we used THP-1 cells for in vitro experiments. The results demonstrated that after activating NLRP3 with LPS & Nig, the expressions of IL-1β and caspase-1 in the supernatant were considerably reduced following EVs treatment (Fig. 4A).

Previous research has revealed that NLRP3 ubiquitination is a critical step in inhibiting NLRP3 activation [47]. As a result, it is hypothesized that hucMSCs-EVs have anti-inflammatory effects via NLRP3 ubiquitination. NLRP3 ubiquitination in distinct groups was further evaluated, but no significant difference was observed in NLRP3 ubiquitination levels between EVs and PBS groups (Fig. 4B). These results suggest that EVs do not have anti-inflammatory properties due to NLRP3 ubiquitination. METTL3-mediated m6A alteration has already been revealed to increase OA development [32]. As a result, a kit was used to identify the m6A level of RNA, and a western blot was used to find METTL3 expression in separate groups following manufacturer's instructions. The results indicated that the level of METTL3 expression in EVs group was much lower than in the PBS group (Fig. 4C), and the level of m6A was also significantly lower (Fig. 4D), demonstrating that hucMSC-EVs may function by inhibiting METTL3 in macrophages.

For validation, METTL3-knockdown macrophages were used to investigate the interaction between EVs and METTL3 in macrophages. The results revealed that after NLRP3 activation by LPS & Nig, the expression level of IL-1β and caspase-1 in the supernatant of METTL3-knockdown macrophages was considerably lowered (Fig. 4E), demonstrating that METTL3 plays a pro-inflammatory role in macrophages. Furthermore, there was no significant difference in inflammatory factors in the supernatant of EVs group compared with PBS group (Fig. 4E), indicating that EVs exhibit anti-inflammatory effects via downregulating METTL3.

### Anti-inflammatory effects of HucMSCs-EVs through miRNA

**Fig. 5:**
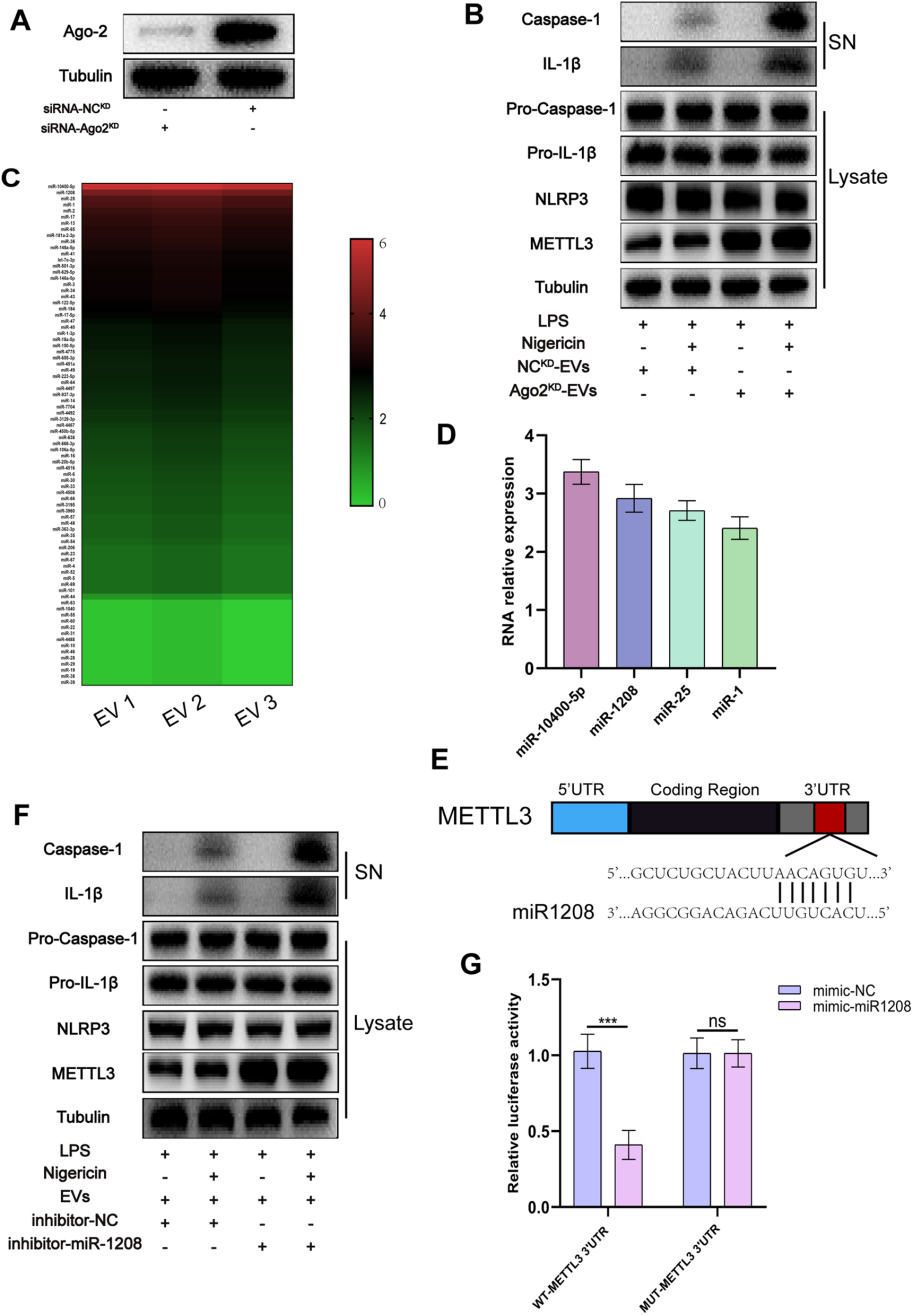
miR-1208 is the key miRNA bind to METTL3 in EVs to exert anti-inflammatory effects. **A.** Western blot analysis of Ago-2 expression in hucMSCs lysates. **B.** Western blot analysis of IL-1β and cleaved caspase-1 levels in culture SN and pro-IL-1β, pro-caspase-1, NLRP3, and METTL3 levels in macrophage cell lysates in each group. **C.** Heat map of miRNA levels in hucMSCs-EVs (*n* = 3, Student’s t test). **D.** Four most significantly upregulated miRNAs in hucMSCs-EVs. **E.** The target sequence of miR-1208 estimated within 3′-UTR of METTL3. **F.** Western blot analysis of IL-1β and cleaved caspase-1 levels in culture SN and pro-IL-1β, pro-caspase-1, NLRP3, and METTL3 levels in macrophage cell lysates in each group. **G.** Luciferase reporter assay showing that METTL3 is the target gene for miR-1208 (n = 3, Student’s t test)

Both in vivo and in vitro investigations revealed that hucMSCs-EVs have an anti-inflammatory impact. However, the main molecules involved in the regulation process require additional investigation. It is well recognized that miRNA plays a vital function in EVs and intercellular communication [48, 49]. The anti-inflammatory actions of hucMSCs-EVs may also be mediated via miRNA. Argonaute 2 (Ago2), a component of RNA-induced silencing complex, is a major regulator of miRNA, either by modulating miRNA-mediated mRNA cleavage activity or blocking translational activity [50]. To obtain Ago2^KD^-EVs, Ago2 in hucMSCs was knocked down using siRNA-Ago2. A Western blot was used to confirm the knockdown impact of Ago2. Western blot analysis revealed a substantial decrease in Ago2 expression following knockdown (Fig. 5A). EVs and Ago2^KD^-EVs were individually introduced to macrophages, and macrophage proteins were isolated and analyzed using western blot. The findings revealed that Ago2^KD^-EVs did not affect the expression of METTL3 or the relative inflammatory factors (Fig. 5B). This displays that miRNAs in hucMSCs-EVs may play a biological role in slowing OA progression.

### miR-1208 may be the key miRNA in EVs to exert anti-inflammatory effects

It is suggested that among various miRNAs in EVs, one specific miRNA may downregulate METTL3 expression and lower m6A levels after interacting with METTL3, hence suppressing NLRP3 activity and lowering inflammatory factor expression. As a result, total RNA from hucMSCs-EVs was isolated and analyzed using microarrays. The miR-10400-5p, miR-1208, miR-25, and miR-1 were highly expressed in hucMSCs-EVs according to the miRNA microarray study (Fig. 5C and D). Furthermore, merging three online databases (TargetScan, PicTar, and DIANA) demonstrated that only miR-1208 could target bind to METTL3 (Fig. 5E). As a result, miR-1208 could selectively bind to METTL3 to perform anti-inflammatory effects.

To further validate the anti-inflammatory effect of miR-1208, it was knocked down in macrophages. Normal macrophages and miR-1208 knockdown macrophages were treated with hucMSCs-EVs, and protein was collected for western blot analysis from each group. The results indicated that miR-1208 suppression did not significantly reduce METTL3 expression in macrophages following EVs treatment and that the anti-inflammatory impact of EVs was decreased (Fig. 5F).

The luciferase reporter assay on 293 T cells was further used to identify if miR-1208 interacts directly with METTL3. According to the results, co-transfection of METTL3 WT (rather than MUT) luciferase construct with miR-1208 mimics lowered luciferase activity (Fig. 5G). This reveals that METTL3 is miR-1208 target gene. The miR-1208 is the primary miRNA in EVs that target METTL3 in macrophages to exhibit anti-inflammatory effects.

### The promotion effect of hucMSCs-EVs on chondrocyte proliferation

**Fig. 6:**
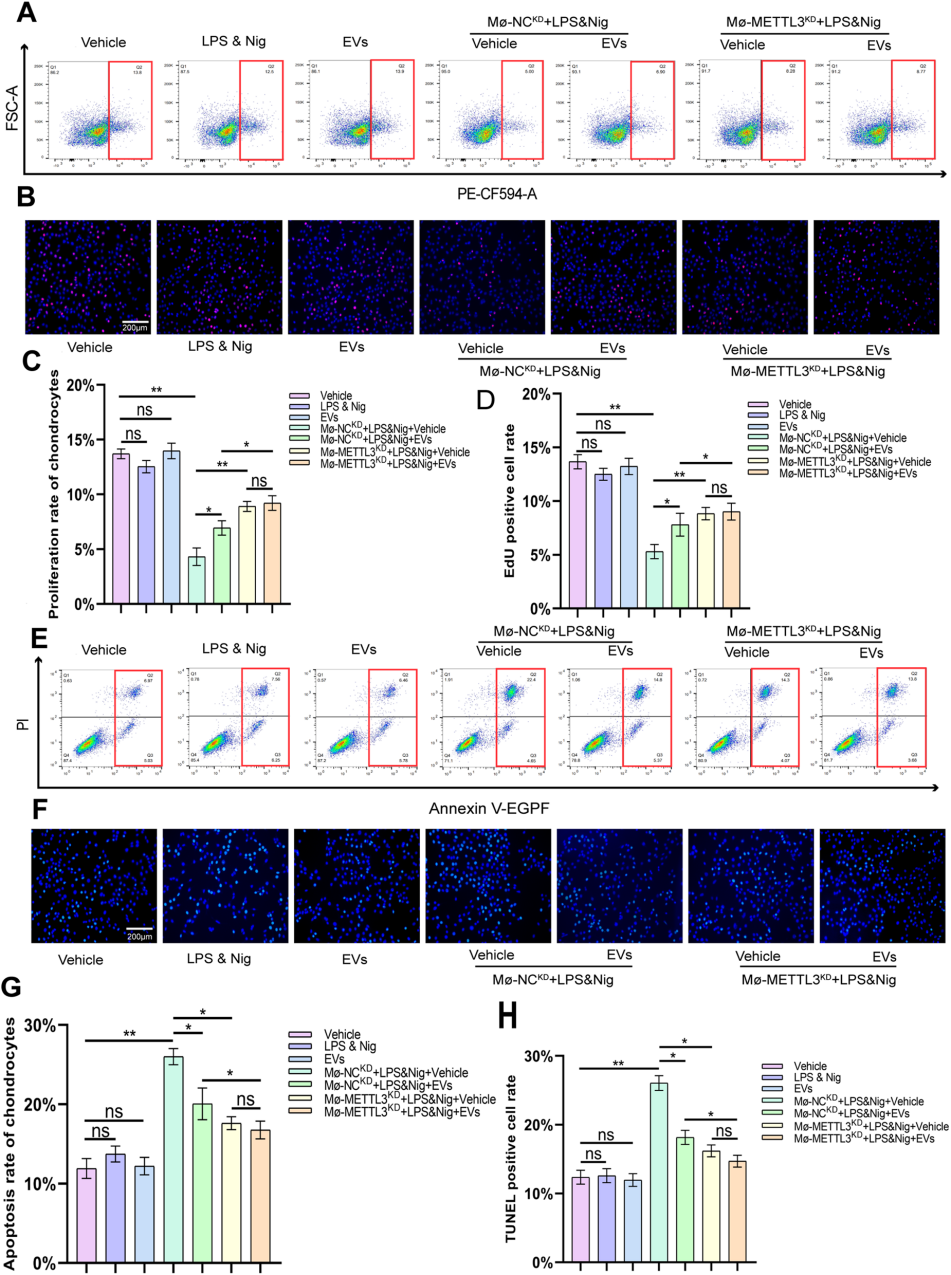
HucMSCs-EVs promote the proliferation and inhibit the apoptosis of chondrocyte by downregulating METTL3 in macrophage. **A.** Proliferation rate of chondrocytes was determined by flow cytometry analysis after EdU assay. **B.** Proliferation rate of chondrocyte as determined by immunofluorescence. Scale bar = 200 μm. **C.** Analysis of flow cytometry results from EdU assay (*n* = 3). **D.** Analysis of EdU positive cell rate in each group (*n* = 3). **E.** The Annexin V-FITC/PI apoptosis assay for apoptosis rate of chondrocytes determined by flow cytometry analysis. **F.** Apoptosis rate of chondrocytes as determined by immunofluorescence. Scale bar = 200 μm. **G.** Analysis of flow cytometry results from the Annexin V-FITC/PI apoptosis assay (n = 3). **H.** Analysis of TUNEL positive cell rate for each group (n = 3). Data are presented as mean ± SD. ns, no significance; *p < 0.05; **p < 0.01; ***p < 0.001

The association of EVs with METTL3 in macrophages was described above, and we then investigated the effect of this interaction on chondrocytes. THP-1 cells and human chondrocytes were co-cultured in the co-culture system. The flow cytometry analysis revealed no statistical change in chondrocyte proliferation rate when co-cultured with the vehicle, LPS & Nig, or EVs. The proliferation rate of chondrocytes was dramatically reduced when co-cultured with macrophages (M_Φ_-NC^KD^) activated by LPS & Nig. The chondrocyte proliferation rate was found to rise after treatment with EVs, and the difference was statistically significant. When METTL3-knockdown macrophages (M_Φ_-METTL3^KD^) were co-cultured with chondrocytes after LPS & Nig stimulation, there was no significant increase in chondrocyte proliferation rate of EVs group compared to the vehicle group (Fig. 6A and C). EdU immunofluorescence results were identical to those of flow cytometry (Fig. 6B and D). These findings suggest that EVs stimulate chondrocyte proliferation via METTL3 in macrophages.

### The inhibitory effect of hucMSCs-EVs on chondrocyte apoptosis

The apoptosis rate of chondrocytes in the co-culture system was determined using the Annexin V-FITC/PI apoptosis assay. The flow cytometry results revealed no significant difference in the apoptosis rate of chondrocytes in the co-culture system among vehicle, LPS & Nig, and EVs groups (Fig. 6E and G). The apoptosis rate of chondrocytes was significantly increased in the co-culture system of macrophages and chondrocytes with LPS & Nig stimulation, whereas the apoptosis rate of chondrocytes was statistically significantly decreased after EVs treatment (Fig. 6E and G). The apoptosis rate of chondrocytes was slightly higher in M_Φ_-METTL3^KD^ co-culture system compared with LPS & Nig group, but the increase was lower than that in M_Φ_-NC^KD^ group and there was no significant decrease in the apoptosis rate after EVs treatment (Fig. 6E and G). The results of TUNEL Green Apoptosis Detection Kit were comparable to those of flow cytometry (Fig. 6F and H). All of this suggests that hucMSCs-EVs inhibit chondrocyte apoptosis via METTL3 in macrophages.

### The promotion effect of hucMSCs-EVs on chondrocyte migration

**Fig. 7:**
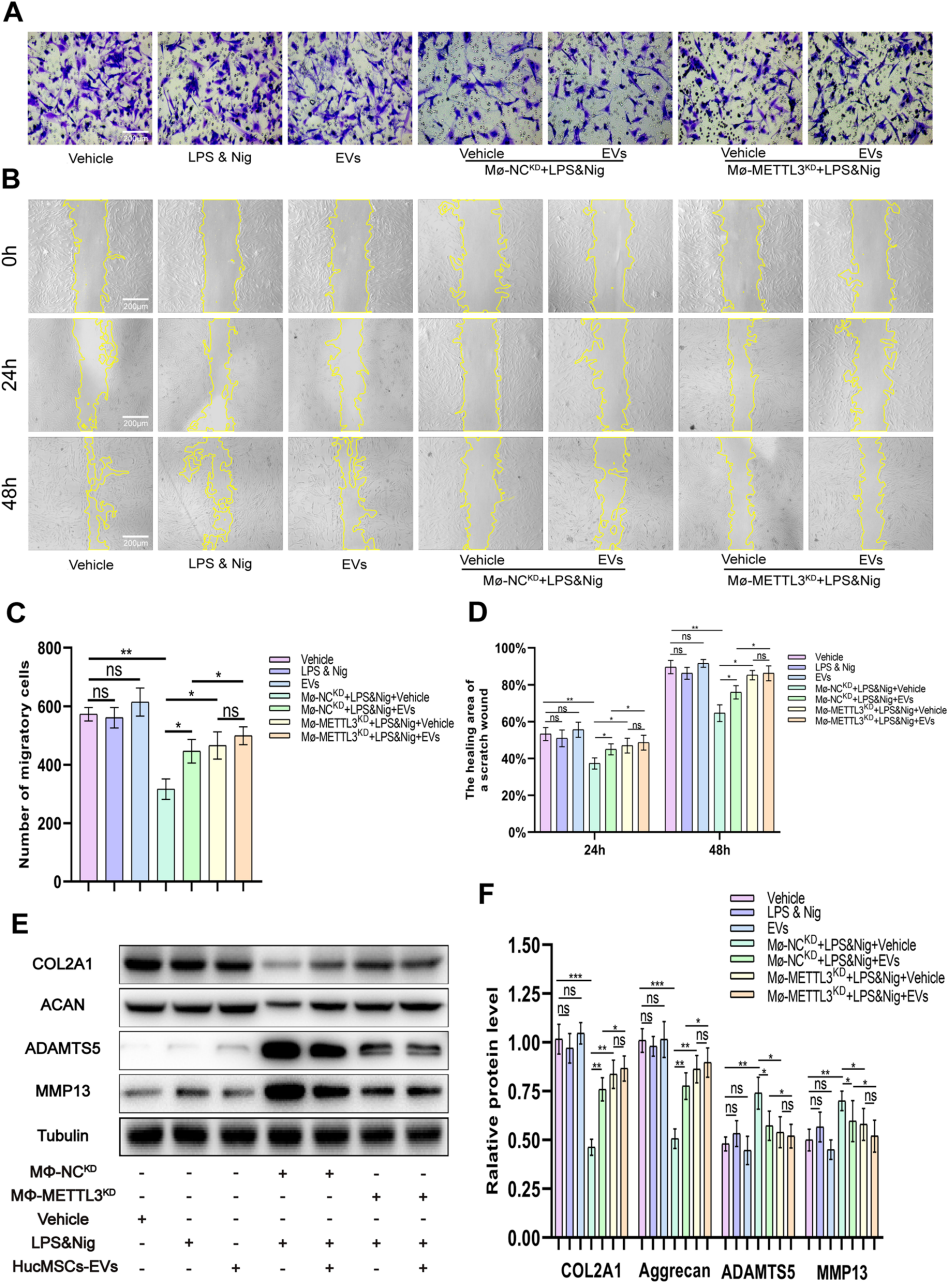
HucMSCs-EVs facilitate the migration of chondrocyte and inhibit the degradation of ECM by downregulating METTL3 in macrophage. **A.** Migration of chondrocytes was observed using transwell assay. Scale bar = 200 μm. **B.** Images of cell scratch area healing assay on chondrocytes were obtained under a light field of microscope at 0 h, 24 h, and 48 h. **C.** The number of migratory cells was calculated and analyzed in each group (*n* = 3). Scale bar = 200 μm. **D.** The healing area of a scratch wound was analyzed in each group (*n* = 3). **E.** Western blot analysis for COL2A1, Aggrecan, ADAMTS5, and MMP13 in each group. **F.** Quantification of the density of immunoreactive bands normalized to tubulin in each group (n = 3). Data are presented as mean ± SD. ns, no significance; *p < 0.05; **p < 0.01; ***p < 0.001

To determine the migration ability of chondrocytes in each group, the transwell assay and cell scratch area healing assay was used. The transwell assay results revealed no statistically significant difference in the number of chondrocyte migration when chondrocytes were cultured with vehicle, LPS & Nig, or EVs (Fig. 7A and C). When chondrocytes were co-cultured with M_Φ_-NC^KD^, the number of chondrocyte migration was significantly reduced with LPS & Nig stimulation, whereas chondrocyte migration was statistically increased after EVs treatment (Fig. 7A and C). When co-cultured with M_Φ_-METTL3^KD^, the decrease in chondrocyte migration was less than that observed in the M_Φ_-NC^KD^ group, and there was no significant increase in chondrocyte migration after EVs treatment (Fig. 7A and C). The cell scratch assay yielded consistent results. EVs treatment improved the healing capacity of chondrocytes co-cultured with M_Φ_-NC^KD^. The ability of EVs to promote chondrocyte healing was lost in M_Φ_-METTL3^KD^ group (Fig. 7B and D). This suggests that EVs facilitate chondrocyte migration via METTL3.

### The protective effect of hucMSCs-EVs on cartilage ECM degradation

Proteins were extracted from chondrocytes in each co-culture system, and the levels of expression of ECM proteins (COL2A1 and Aggrecan) and ECM protein-degrading enzymes (ADAMTS5 and MMP13) were determined using western blot. The results revealed no significant difference in the expression levels of COL2A1, Aggrecan, ADAMTS5, and MMP13 among vehicle group, LPS & Nig group, and EVs group (Fig. 7E and F). However, when M_Φ_-NC^KD^ and chondrocytes were co-cultured with LPS & Nig, the expression of ECM proteins was significantly reduced, while the expression of ECM protein-degrading enzymes was significantly increased (Fig. 7E and F). While COL2A1 and Aggrecan levels statistically recovered after EVs treatment, ADAMTS5 and MMP13 expression levels significantly decreased (Fig. 7E and F). However, the protective effect of hucMSCs-EVs was lost in M_Φ_-METTL3^KD^ group, as there was no significant difference in the increase in COL2A1 and Aggrecan levels and the decrease in ADAMTS5 and MMP13 expression following EVs treatment (Fig. 7E and F). These findings suggest that hucMSC-EVs inhibit COL2A1 and Aggrecan degradation and counteract ADAMTS5 and MMP13 elevation via METTL3 in macrophages.

### Depletion of miR-1208 in vivo inhibits the cartilage protective and matrix protein regulation effects of hucMSCs-EVs

**Fig. 8:**
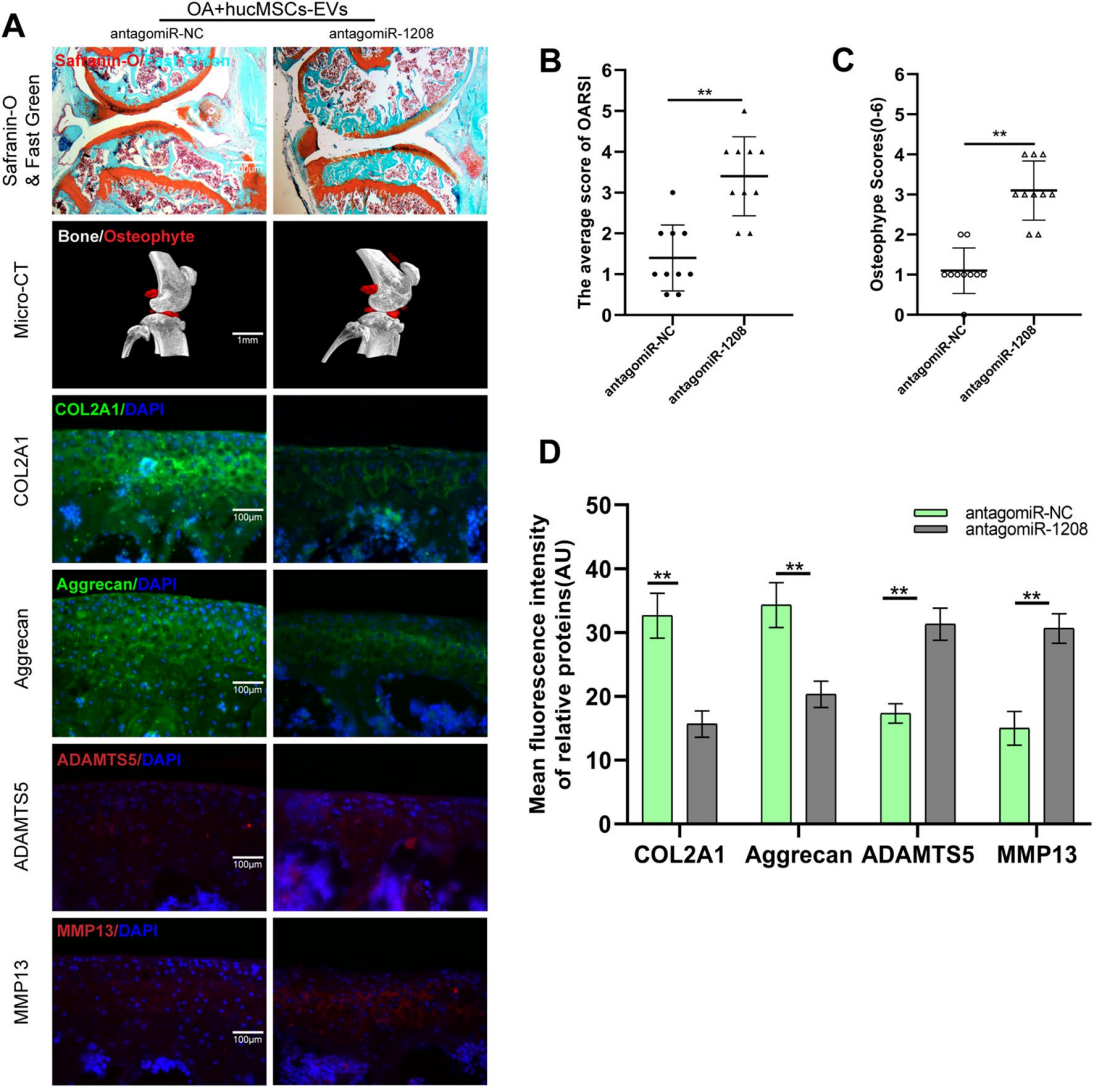
miR-1208 alleviates knee osteoarthritis and decreases the cartilage degradation in vivo. **A.** Safranin O and Fast Green, Micro-CT, and immunofluorescence staining of each group of mice knee section. **B.** OARIS scores of knee sections in each group (*n* = 10). **C.** Osteophyte score of each group (*n* = 10). **D.** Quantification of mean fluorescence intensity of COL2A1, Aggrecan, ADAMTS5, and MMP13 in each group (*n* = 10)

To further investigate the role of miR-1208 in EVs-mediated chondroprotection in vivo in a mice model of DMM, antagomiR-1208 was used. Safranin O and Fast Green were used to stain sample sections. The results indicated that the miR-1208 knockout mice's cartilage was severely damaged, and their OARSI score was significantly higher than that of the miR-NC knockout mice (Fig. 8A and B). Mice knee micro-CT images and osteophyte scores revealed a significant increase in mice knee osteophytes in the presence of antagomiR-1208 (Fig. 8A and C). EVs did not inhibit the degradation of COL2A1 and Aggrecan, nor the expression of ADAMTS5 and MMP13, as determined by immunofluorescence staining of sample sections in the miR-1208 knockout OA mice model (Fig. 8A and D). These findings suggest that hucMSCs-EVs inhibit the expression of ADAMTS5 and MMP13 and the degradation of COL2A1 and Aggrecan in OA mice models via miR-1208.

## Discussion

The most common site of OA is the knee [2], which is characterized by articular cartilage degeneration, space narrowing, and osteophyte formation [2, 6]. Inflammation is a significant factor in OA progression [6, 9]. Macrophages that secrete pro-inflammatory factors such as IL-1β and IL-18 are critical in the development of knee OA [17]. Pro-inflammatory factors such as IL-1β induce chondrocyte apoptosis and disrupt the knee joint's homeostasis [51, 52]. Additionally, it can accelerate the degradation of COL2A1 and Aggrecan, as well as the production of ADAMTS5 and MMP13, further destroying the knee cartilage [53]. As a result, the key to treating OA is to control inflammation.

Recent years have demonstrated a surge of interest in research on MSCs therapy for degenerative diseases [54, 55]. However, MSCs have limited clinical applications due to their adverse effects, such as possibility of chromatin variations and immune rejection, which could lead to many adverse consequences [36]. MSCs primarily function via the pathway, according to research [56]. As a result, EVs have garnered considerable attention as a mode of intercellular communication [40, 57]. When recipient cells endocytose EVs, the genetic information carried by miRNAs contained within them affects the biological activity and related protein expression of these cells. EVs from bone marrow MSCs and synovium MSCs have recently been demonstrated to help prevent osteoarthritis development [54, 58, 59]. Due to the ease of extraction and great differentiation potential of hucMSCs, hucMSCs-EVs have much potential for treating OA [57, 60].

This study discovered that hucMSCs-EVs could significantly alleviate symptoms in a DMM-induced knee OA mouse model, alleviate articular cartilage degeneration, alleviate COL2A1 and Aggrecan degradation, and inhibit ADAMTS5 and MMP13 expression. Additionally, it can significantly inhibit the formation of knee osteophytes and the production of pro-inflammatory factors such as IL-1β from reducing knee joint inflammation, thus helping in delaying knee OA development.

Macrophages are involved in a variety of inflammations [61, 62]. Macrophages have been linked to cartilage regeneration and may be used to protect the cartilage ECM from degradation [62, 63]. Additionally, macrophages contribute significantly to the stability of knee microenvironment [62]. As a result, when THP-1 cells were co-cultured with chondrocytes, the macrophages secreted more inflammatory factors after LPS and Nig stimulation, inhibited chondrocyte proliferation and migration, and increased chondrocyte apoptosis. NLRP3 is an important component of macrophage inflammation [20, 47], which has been identified as a potential novel biomarker for OA linked to the induction of IL-1β and IL-18 secretion, both of which promote cartilage degeneration and synovial inflammation [23, 47]. As a result, we used NLRP3^−/−^ mice and discovered that the protective effect of hucMSCs-EVs on knee OA was abolished, implying that the EVs play a role via NLRP3. As one of the most common post-transcriptional RNA modifications, m6A accounts for more than 80% of all RNA methylation modifications and is involved in various physiological activities such as aging, degeneration, and inflammation [25, 27, 32, 64]. In our study, an increased level of m6A modification in OA was discovered, accompanied by an increase in METTL3 expression. Because METTL3 is a key component of the m6A methyltransferase, an increase in METTL3 results in an increase in m6A RNA levels. Our study further observed that hucMSCs-EVs reduced m6A levels in RNA by inhibiting METTL3 expression, lowering NLRP3 activity, inhibiting inflammation, and slowing OA progression.

It is widely acknowledged that EVs are an essential means of information communication between cells [49] and that they function primarily through the miRNAs contained within them [48, 49]. The next step was to investigate miRNA mechanism by which hucMSCs-EVs alleviate OA, where miR-1208 was found to inhibit METTL3 expression and reduce the m6A level of mRNA and NLRP3 activity, thereby exerting an anti-inflammatory effect and delaying OA which was confirmed using several experimental and gene prediction websites. Indeed, there are also shortcomings in this study. Although in vitro we used THP-1 cells for mechanistic studies, the impact of macrophages on chondrocytes in the native joint need further research. We gave the mice intra-articular injection of antagomiR three weeks before surgery according to the reported literature, but the optimal injection time still needs further study. In the study of ECM, we only take classical COL2A1 and Aggrecan into consideration. Recent studies have shown that heparan sulfate also plays an important role in cartilage ECM [65], which needs further study. Furthermore, it was predicted that hucMSCs-EVs work through changes in protein content within them, but this was put on hold due to resource constraints and required further investigation. Mice in the sham group and NLRP3 ^−/−^ mice also developed early OA due to excessive exercise, and EVs could only partially alleviate OA. Further exploration is needed to completely cure OA.

## Conclusion

**Fig. 9:**
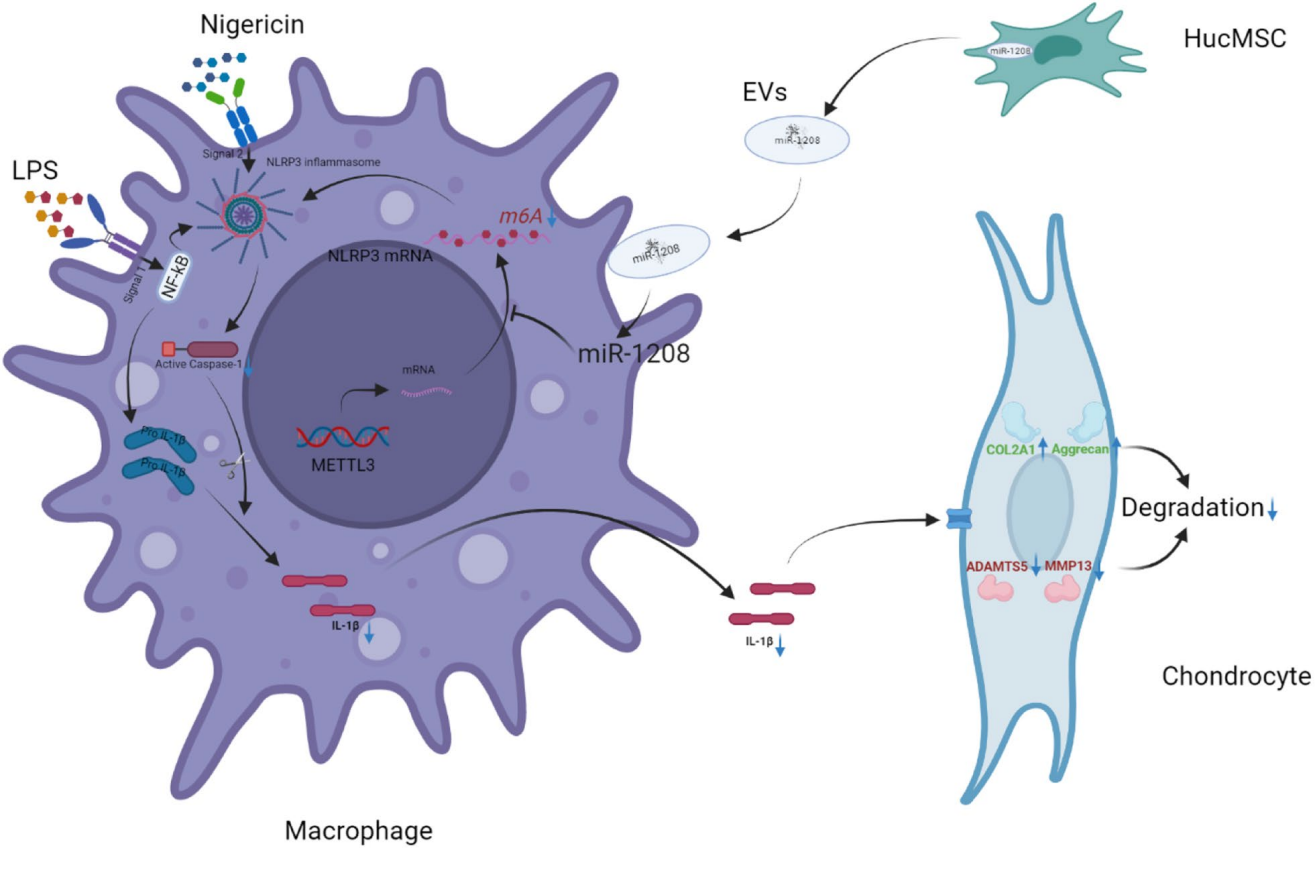
Model of miR-1208 within hucMSCs-EVs targeting METTL3 in macrophages prevents the secretion of inflammatory cytokines by regulating the activation of NLRP3 and the degradation of chondrogenic ECM

This study found that hucMSCs-EVs can reduce OA occurrence and progression, promote chondrocyte proliferation and migration, and inhibit chondrocyte apoptosis. miR-1208 in EVs could target METTL3 to reduce NLRP3 mRNA methylation, suppress inflammatory factor release, inhibit COL2A1 and Aggrecan degradation, and inhibit ADAMTS3 and MMP13 (Fig. 9). EVs significantly alleviated OA development in DMM-induced mice OA model and prevented OA from occurring.

## Data Availability

All relevant data and materials are available from the authors upon reasonable request.

## Abbreviation

HucMSCs-EVs: Extracellular vesicles derived from human umbilical cord mesenchymal stem cells
MSCs: Mesenchymal stem cells
OA: Osteoarthritis
m6A: N6-methyladenosine
METTL3: Methyltransferase-like 3
NLRP3: Nod-like receptor pyrin domain 3 inflammasomes
DMM: Destabilization of the medial meniscus
COL2A1: Collagen type II alpha 1
ECM: Extracellular matrix
ADAMTS5: A disintegrin-like and metalloproteinase domain with thrombospondin-1 motifs 5
MMP13: Matrix metalloproteinase 13
PBS: Phosphate-buffered saline
miRNAs: MicroRNAs
siRNA: Small interfering RNA
TKA: Total knee arthroplasty
DMEM/F12: Dulbecco’s Modified Eagle Medium/Nutrient Mixture F-12
FBS: Fetal bovine serum
P/S: Penicillin/streptomycin
TEM: Transmission electron microscope
DAPI: 4′,6-Diamidino-2-phenylindole
ANOVA: Analysis of variance
OARSI: Osteoarthritis research society international
Ago2: Argonaute 2
